# The Possible Neuronal Mechanism of Acupuncture: Morphological Evidence of the Neuronal Connection between Groin A-Shi Point and Uterus

**DOI:** 10.1155/2013/429186

**Published:** 2013-02-28

**Authors:** Chun-Yen Chen, Rey-Shyong Chern, Ming-Huei Liao, Yung-Hsien Chang, Jung-Yu C. Hsu, Chi-Hsien Chien

**Affiliations:** ^1^Department of Cell Biology and Anatomy, College of Medicine, National Cheng-Kung University, Tainan, Taiwan; ^2^Graduate Institute and Department of Veterinary Medicine, College of Veterinary Medicine, National Pingtung University of Science and Technology, 1, Shuefu Road, Neipu, Pingtung 912, Taiwan; ^3^Graduate Institute of Chinese Medical Science, China Medical University, Taichung, Taiwan

## Abstract

Somatovisceral reflex suggested that the somatic stimulation could affect visceral function like acupuncture which treats diseases by stimulating acupoints. The neuronal connection between somatic point and visceral organ was not clear. Uterine pain referred to the groin region has long been recognized clinically. Wesselmann, using neurogenic plasma extravasation method, showed that uterine pain was referred to the groin region through a neuronal mechanism (Wesselmann and Lai 1997). This connection could be considered through the somatovisceral reflex pathway. However, the relay center of this pathway is still not clearly identified. In the present study, bee venom was injected in the groin region to induce central Fos expression to map the sensory innervation of groin region. Pseudorabies virus (PrV), a transneuronal tracer, was injected in the uterus to identify the higher motor control of the uterus. Immunohistochemistry staining revealed the Fos expression and PrV-infected double-labeled neurons in the nucleus of solitary tract (NTS), the dorsal motor nucleus of vagus (DMX), and the paraventricular hypothalamic nucleus (PVN). These results suggest a somatoparasympathetic neuronal connection (groin-spinal dorsal horn-NTS/DMX-uterus) and a somatosympathetic neuronal connection (groin-spinal dorsal horn-NTS-PVN-uterus). These two neuronal connections could be the prerequisites to the neuronal basis of the somatovisceral reflex and also the neuronal mechanism of acupuncture.

## 1. Introduction 

The somatovisceral reflex was mentioned by Sato in 1995 and suggested that somatic stimulation could evoke sympathetic reflex response and, thereby, modulate functioning of visceral organ [1]. This phenomenon is in some way alike acupuncture that stimulates specific somatic points to relieve pain and treat many different diseases [2]. Many studies have shown that acupuncture can significantly modulate visceral function by stimulating specific acupoints [3–8]. Previous research suggested that the activation of the somatosensory pathway played an important role in the physiological effects of acupuncture [9]. Li et al.'s research showed that electroacupuncture-like stimulation diminishes regional myocardial ischemia triggered by sympathetical excitation [7]. Other studies have shown that electroacupuncture-like stimulation can activate a sympathetic inhibitory system in the brain to regulate cardiovascular responses [5, 10, 11]. Both the somatovisceral reflex and acupuncture stimulation suggest the neuronal connection between somatic acupoint and its corresponding organ. However, the neuronal connection of the somatovisceral reflex or acupuncture is still not clear.

Pervious report demonstrated that gynecological pain induced by dysmenorrhea, ascending genital infection, or cystic or hemorrhagic ovarian pathology usually refer pain to the low back, thighs, and abdominal wall [12]. Referred pain in the low back and abdominal wall was also reported by women in labor [13]. These reports suggested that the groin region can account to the pain of uterine inflammation or diseases. According to traditional Chinese medicine, some acupoints, called A-shi points, do not have fixed specific locations and are usually pain-associated points [14–16]. Therefore, the groin region could be the A-shi point related to the uterus. In 1997, Wesselmann and Lai found that uterine inflammation in rats pretreated with Evans Blue Dye resulted in neurogenic plasma extravasation of dye in the skin over the abdomen, lower back, thighs, and groin, after antidromic stimulation of peripheral nerves [17]. This result suggested the possibility of a somatovisceral neural connection between the uterus and groin areas. Although these findings confirm the existence of a neural connection between the uterus and groin region, the exact location of this central neuronal connection remains unknown.

The Fos protein is an immediate-early gene transcription factor induced by short-term signals and alters target gene expression causing long-term change in cellular phenotype [18]. It has been used to map the activated neural cells after different types of stimulation and shows correlated anatomical neural pathways [19–21]. Pseudorabies virus (PrV) is a swine neurotropic herpes virus that has been used for transneuronal tracing in many studies [22–26]. The Pingtung (PT) strain of PrV has been demonstrated to label sympathetic pre- and postganglionic neurons after injection in the specific auricular kidney point [22]. The study showed that the PT strain of PrV was a useful transneuronal tracer in somatovisceral research. To establish the neural connection between the groin region and uterus, bee venom was injected in the groin region to induce c-Fos expression neurons innervating the groin region and PrV was injected in the uterus to infect the hierarchical motor neurons innervating the uterus. Furthermore, to evaluate central doubled Fos expression and PrV-infected neurons in order to identify the neuronal connection between the somatic point (groin region) and its related visceral organ (the uterus).

## 2. Materials and Methods

The study protocol was approved by Animal Care and Use Committee, and all experiments were conducted in accordance with the animal care guidelines of the National Institutes of Health and the International Association for the Study of Pain.

### 2.1. Animals

Sprague-Dawley adult virgin female rats (250–350 g) were used. Animals were housed on a 12 h-12 h light-dark cycle, and all animals had free access to standard food and water. 

### 2.2. Bee Venom Injection in the Left Groin Region

The rats were anesthetized with ketamine (95 mg/kg) intraperitoneally. 50 *μ*L of 1% bee venom (Sigma) were dissolved in normal saline and administrated subcutaneously into the midpoint between genital pore and apex of the left groin region (*n* = 6) according to Wesselmann and Lai's research [17]. Saline was injected as the control. After 90 minutes, the rats were sacrificed and perfused with 250 mL of saline intracardially, followed by 1000 mL of 4% paraformaldehyde in 0.1 M phosphate buffer solution (PBS). T10–S1 segments of spinal cord, brainstem, and brain were removed. 

### 2.3. Pseudorabies Virus Injection in Left Uterine Horn

The rats were anesthetized with ketamine (1 mL/kg) intraperitoneally. A laparotomy was performed and 40 *μ*L of Pingtung strain pseudorabies virus [22] was injected into the left uterus horn (*n* = 9). After the injections, the abdominal wall was sutured, the skin closed. The animals were sacrificed at 6 to 8 days after PrV injection (in the same way as described above). The spinal cord, dorsal root ganglion of T10–S2 segment, brainstem, and brain were removed.

### 2.4. Bee Venom Injection after PrV Injection

The rats were anesthetized and PrV was injected into the left uterus horn (in the same way as described above) (*n* = 9). After the injections, the abdominal wall was sutured, the skin closed, and the animals allowed to survive for 6 to 8 days. Before the rats were sacrificed, 50 *μ*L of 1% bee venom was administrated subcutaneously into midpoint of left groin region and saline was injected as the control. After 90 minutes, the rats were sacrificed and perfused with 250 mL of saline intracardially, followed by 1000 mL of 4% paraformaldehyde in 0.1 M phosphate buffer solution (PBS). The spinal cord, dorsal root ganglion of T10–S1 segment, brainstem, and brain were removed.

### 2.5. Immunohistochemistry

Tissues of groin region-bee venom injection group and uterine horn-pseudorabies virus injection group were postfixed up to 4 hr in paraformaldehyde PBS and then cryoprotected in 10, 20, and 30% sucrose in PB solution. Serial 30 *μ*m thick transverse sections of all dorsal root ganglia, spinal cord, brainstem, and brain were cut with a cryomicrotome. All sections from the ganglia and every five sections from other samples were collected in 0.01 M phosphate buffer saline (PBS). Floating sections were washed 30 min (10 min, 3 times) and incubated with blocking solution (5% normal goat serum, 0.05% Triton X-100, and 3% BSA in 0.1 M PB) for 1 hr. The sections were washed and incubated with the primary antibody (IgG of rabbit anti-FOS in 1 : 2000 or IgG of swine anti-PrV in 1 : 1000) in blocking solution for 72 hr at 4°C. After incubation, the sections were rinsed and incubated for 1 hr at 25°C with secondary antibody (biotin-conjugated IgG of goat anti-rabbit in 1 : 500 or goat anti-swine in 1 : 200) in blocking solution. The sections were washed three times for 30 min and incubated using ABC kit (Vector) for 1 hr. After rinsing, the sections were developed with GOD method followed by mounting on gelatin-coated slides and overslipped with mounting medium.

### 2.6. Immunofluorescence

Tissues of groin-uterus injection group were postfixed and sectioned in the same way described above. Floating sections were washed 30 min (10 min, 3 times) and incubated with blocking solution (5% normal goat serum, 0.05% Triton X-100, and 3% BSA in 0.1 M PB) for 1 h. The sections were washed and incubated with two kinds of primary antibody (IgG of rabbit anti-FOS in 1 : 2000 and IgG of swine anti-PrV in 1 : 1000) in blocking solution for 72 hr at 4°C. After incubation, the sections were rinsed and incubated for 1 hr at 25°C with two secondary antibodies (FITC-conjugated IgG of goat anti-swine in 1 : 200 and TRITC-conjugated IgG of goat anti-rabbit in 1 : 500) in blocking solution. The sections were washed for 30 min and mounted on gelatin-coated slides followed by coverslipping with mounting medium.

### 2.7. Data and Statistical Analysis

Fos and PrV immunoreactivity neurons developed with GOD method in dorsal root ganglia, spinal cord, and brain were counted with bright field microscope. Fos and PrV double labeled neurons were observed with fluorescent microscope. Anatomical identification of brain structures was essentially based on the Rat Brain atlas of Paxinos and Watson [27]. All data were analyzed by *t*-test.

## 3. Result

### 3.1. Fos Expression Neurons after Bee Venom Stimulation in the Groin Region

**Figure 1:**
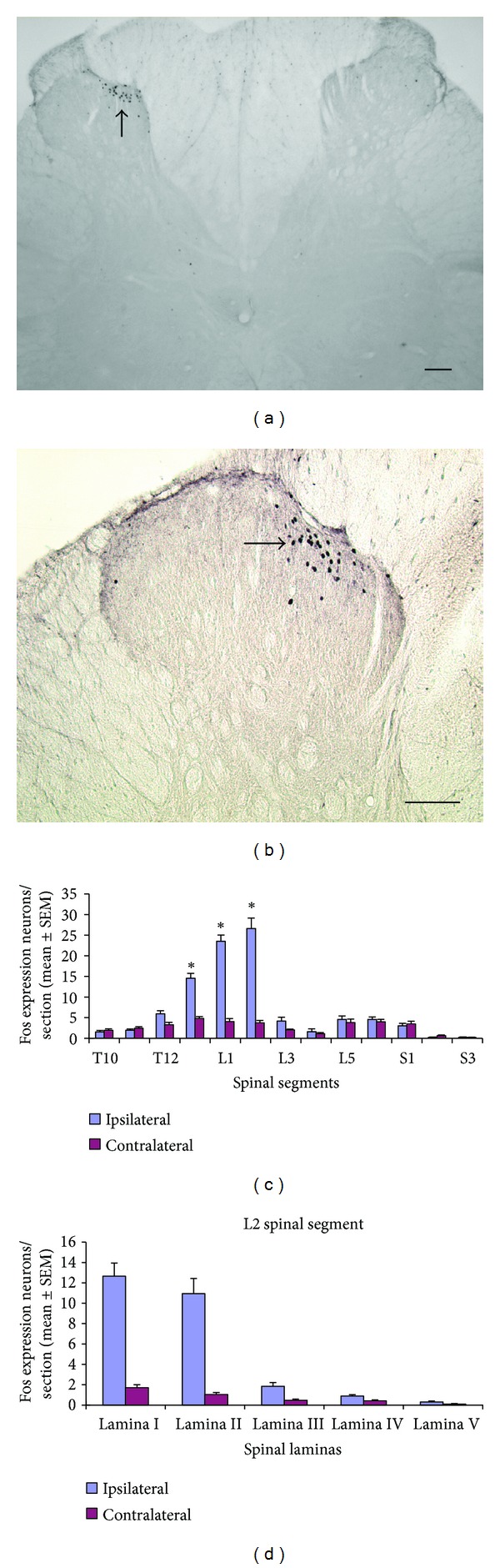
Fos expression neurons in the spinal cord after bee venom injection in the left groin region (*n* = 9). (a) Neurons express Fos protein (arrow) in ipsilateral L2 spinal dorsal horn. (b) Higher magnification of Fos expression neurons in (a) (scale bar: 100 um). (c) Mean number of Fos expression neurons in T10 to S3 spinal segments (±SEM). *P < 0.05. (d) Mean number of Fos expression neurons in laminas I to V of L2 spinal segment.

Injecting bee venom in the left somatic groin region induces central Fos expression and the contralateral side as the control. In the spinal cord, Fos protein is predominantly (70%) apparent in ipsilateral T13 (14.5 ± 1.1), L1 (23.5 ± 1.5), and L2 (26.6 ± 2.5) spinal dorsal horn (Figures 1(a), 1(b), and 1(c)). Most of the c-Fos expression neurons are resided in laminas I (12.6 ± 1.2) and II (10.9 ± 1.4) of the dorsal horn (Figure 1(d)). 

**Figure 2:**
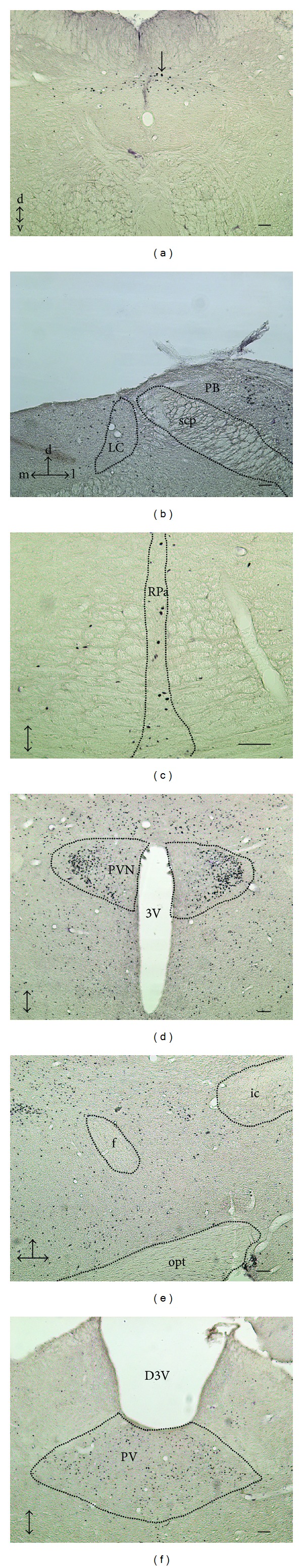
Fos expression neurons (arrow) in the supraspinal area after bee venom injection in the left groin region (*n* = 9). (a) Nucleus of solitary tract (NTS). (b) Locus coeruleus (LC), parabrachial nucleus (PB). (c) Raphe pallidus nucleus (RPa). (d) Paraventricular hypothalamic nucleus (PVN). (e) Lateral hypothalamic area. (f) Paraventricular thalamic nucleus (PV). 3V: third ventricle; 4V: fourth ventricle; D3V: dorsal third ventricle; d: dorsal; f: fornix; ic: internal capsule; l: lateral; m: medial; opt: optic tract; scp: superior cerebellar peduncle; v: ventral (scale bar: 100 um).

In the supraspinal area, c-Fos expression neurons appeared in numerous nuclei of the thalamus, hypothalamus, pons, and medulla. The c-Fos expression nuclei include the nucleus of solitary tract (NTS) (Figure 2(a)), parabrachial nucleus (PB), locus coeruleus (LC) (Figure 2(b)), raphe pallidus nucleus (RPa) (Figure 2(c)), paraventricular thalamic nucleus (PVT) (Figure 2(f)), lateral hypothalamic area (LH) (Figure 2(e)), and paraventricular hypothalamic nucleus (PVN) (Figure 2(d)).  Table 1 listed the fos expression neurons in supraspinal areas of saline and bee venom groups. The NTS of bee venom group expressed significantly the difference between the fos expression neurons and the saline group.

### 3.2. The Appearance of PrV Infection Neurons after Virus Injection in the Uterus

**Figure 3:**
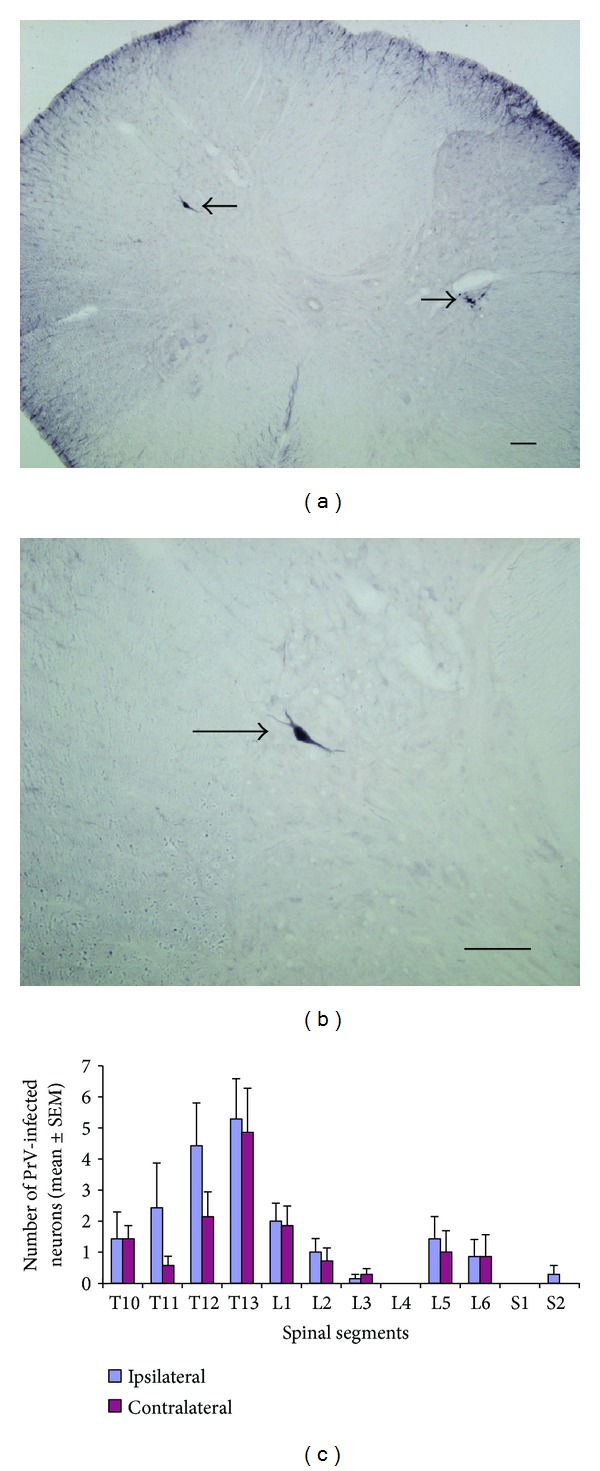
PrV-infected neurons in spinal cord after 6 to 8 d PrV injection in the left uterine horn (*n* = 8). (a) PrV-infected neuron (arrow) in T12 spinal segment. (b) Higher magnification of PrV-infected neuron in (a) (arrow) (scale bar: 100 um). (c) Mean number of PrV-infected neurons in T10 to S2 spinal segments (±SEM).

**Figure 4:**
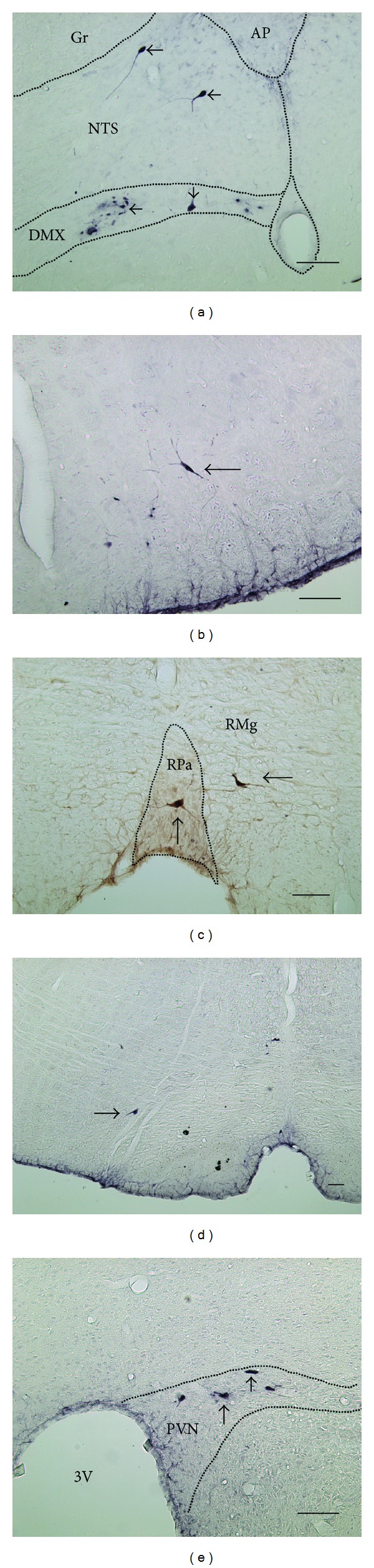
PrV-infected neurons (arrow) in the supraspinal area after 7-8 d PrV injection in the left uterus horn (*n* = 8). (a) PrV-infected neurons in the NTS and DMX. (b) PrV-infected neurons in the A5 noradrenaline cell group. (c) PrV-infected neurons in the RPa and RMg. (d) PrV-infected neurons in the Gi. (e) PrV-infected neurons in the PVN. 3V: third ventricle; AP: area postrema; DMX: motor nucleus of vagus; Gi: gigantocellular reticular nucleus; Gr: gracile nucleus; NTS: nucleus of solitary tract; PVN: paraventricular hypothalamic nucleus; RPa: raphe pallidus nucleus; RMg: raphe magnus nucleus (scale bar: 100 um).

PrV-infected neurons appeared in the central nuclei (Figures 3 and 4) 6–8 days after PrV injection in the left uterus horn. In the spinal cord, the most PrV-infected neurons were spotted in laminas IV and V of T11–L1 and L5–S2 spinal segments (Figures 3(a), 3(b), and 3(c)), rarely in the superficial laminas (laminas I, II). 

In the supraspinal area, PrV-infected neurons were found in the hypothalamus, pons, and medulla, including the NTS, dorsal motor nucleus of vagus (DMX) (Figure 4(a)), intermediate reticular nucleus (IRt), ambiguus nucleus (Amb), lateral reticular nucleus (LRt), A5 noradrenaline cell group (A5) (Figure 4(b)), raphe pallidus nucleus (RPa) (Figure 4(c)), gigantocellular reticular nucleus (Gi) (Figure 4(d)), medial preoptic area (MPA), and PVN (Figure 4(e)). All PrV-infected neurons in supraspinal area were listed in Table 2.

### 3.3. Fos Expression and PrV-Infected Double Labeled Neurons in the NTS, DMX, and PVN

**Figure 5:**
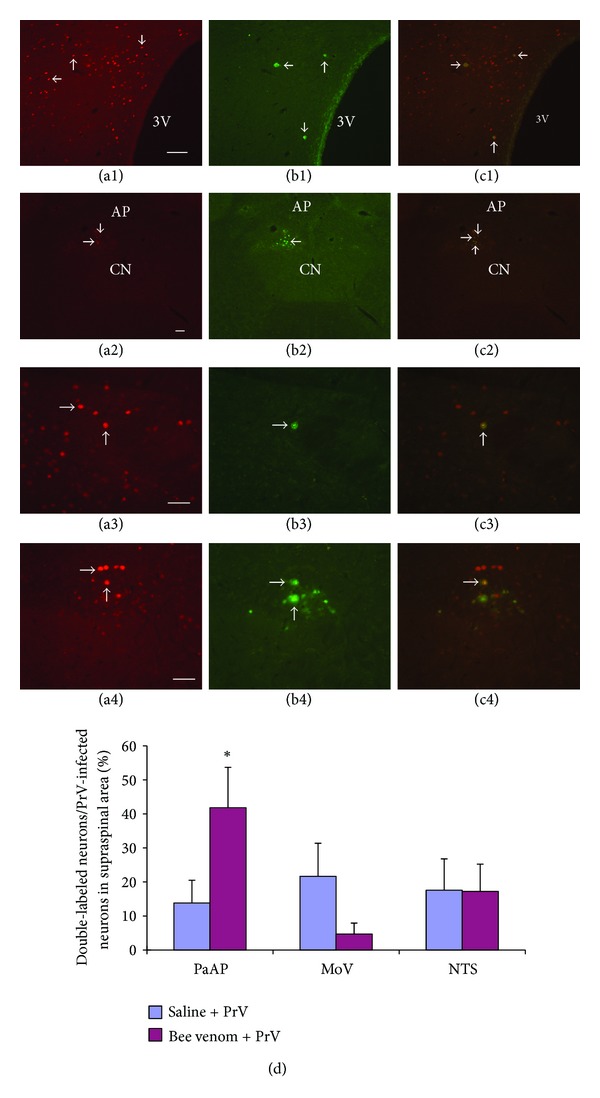
Double-labeled neurons of Fos expression and PrV infection (*n* = 8). Fos expression neurons ((a), red, arrow) in the PVN (a1), NTS ((a2), (a3)), and DMX ((a2), (a4)). PrV-infected neurons ((b), green, arrow) in the PVN (b1), NTS ((b2), (b3)), and DMX ((b2), (b4)). Merged double labeling Fos expression and PrV-infected neurons ((c), yellow, arrow) in the PVN (c1), NTS ((c2), (c3)), and DMX ((c2), (c4)) (scale bar: 1000 um). (d) The percentage of double labeled neurons in PVN, DMX, and NTS between saline and bee venom injection groups (*P < 0.1).

After uterine PrV injection and c-Fos expression of groin bee venom stimulation, double labeled neurons appeared in the hypothalamus, and specifically in the PVN (Figure 5(c1)). Some other double labeled neurons are apparent in the NTS and DMX (Figures 5(c2), 5(c3), and 5(c4)). Comparing saline and bee venom injection groups in PrV-infected neurons, the percentage of the double labeled neurons in PVN of bee venom injection group were significantly predominant (*P* < 0.1) (Figure 5(d)).

## 4. Discussion

After the application of bee venom to the left groin region, the c-Fos protein expression neurons were presented in the left spinal dorsal horn and certain supraspinal nuclei. PrV-infected uterine supraspinal neurons resided in the A5 noradrenaline cell group (A5), raphe pallidus nucleus (RPa), and gigantocellular reticular nucleus (Gi). Double labeled neurons located in the NTS, motor nucleus of vagus (DMX), and PVN (Figure 5). The neuronal connection between groin region and uterus suggests that the nuclei of PVN, NTS, and DMX not only receive somatic stimulation from groin region but also modulate the function of uterus. Those nuclei may play important roles in somatovisceral reflex [1] and could be the result of the neuronal mechanism of acupuncture.

### 4.1. Sensory Innervation of the Left Groin A-Shi Point

The stimulating acupoints elicit a composite of unique sensations called* dechi*. *Dechi* sensations including pressure, soreness, heaviness, and dull pain are essential for clinical efficacy [28]. Pain, as one of the *dechi* sensations, is a relatively strong and easily induced sensory modality in animal study. Panic stimulation can induce neurons expressing the Fos protein which could be used to investigate either somatic [29–31] or visceral [31–33] noxious afferent pathways. Takahashi and Nakajima [34] intravenously injected in the Evans Blue Dye and observed extravasation in the groin after electrical stimulation of the spinal nerves. This result proves that the sensory innervation of groin region comes from T13, L1, and L2 spinal nerves. The injection of bee venom in the left groin region in our study induced c-Fos expression in the ipsilateral spinal dorsal horn of T13, L1, and L2, and particularly in superficial laminas I and II (Figures 1(c) and 1(d)). This suggests that the spinal Fos expression neurons in our study were specifically activated by noxious stimulation of the groin region.

**Figure 6:**
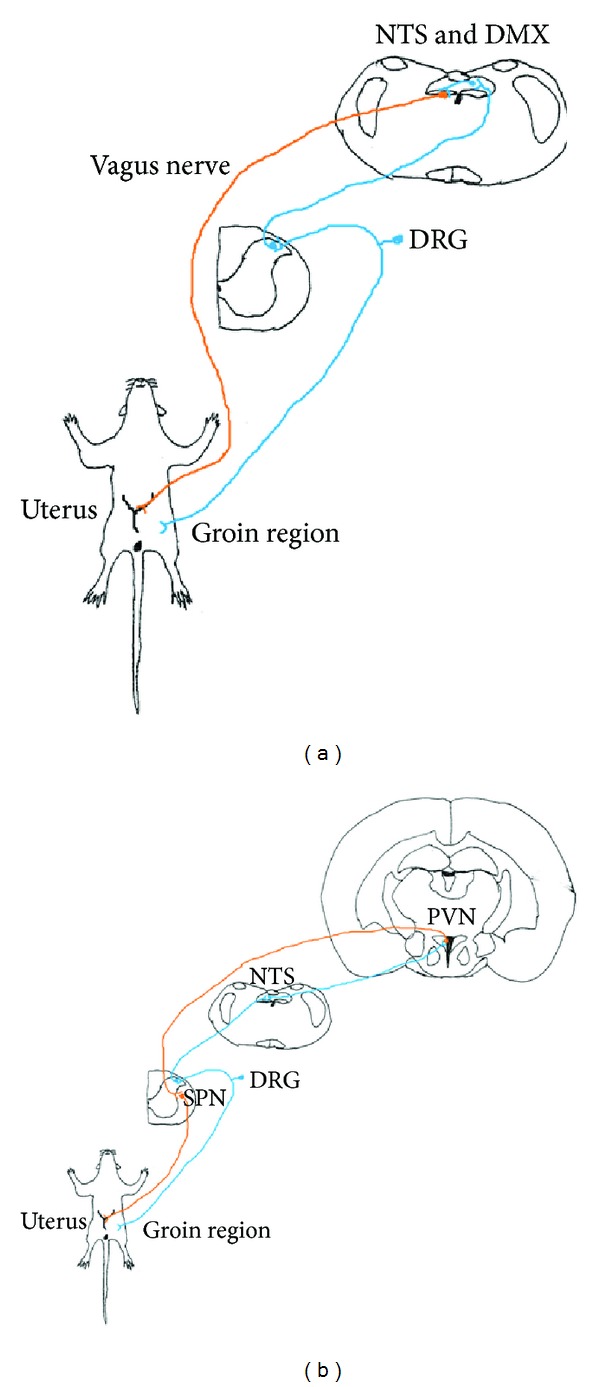
Schematic drawing of three neuronal pathways of somatovisceral reflex. (a) Somato-parasympathetic reflex pathway through the vagus nerve. (b) Somato-sympathetic reflex pathways through the NTS and PVN. DMX: dorsal motor nucleus of vagus; DRG: dorsal root ganglia; NTS: nucleus of solitary tract; SPN: sympathetic preganglionic neurons; PVN: paraventricular hypothalamic nucleus; blue: somatic afferent; red: visceral efferent.

Fos expression neurons appeared in the NTS, gigantocellular reticular nucleus (Gi), raphe pallidus nucleus (RPa), and PVN after bee venom stimulated groin region (Figures 3 and 6). The NTS can be not only activated by somatic noxious stimulation but also triggered by vagal afferent activation as a physiological adaptation to pain [35]. Previous studies showed that Gi can be activated by noxious stimulation related to the activation of the descending antinociceptive pathway [36–39]. Electrically stimulating the raphe nucleus could induce analgesia, proving that the raphe nucleus plays a crucial role in pain inhibition response [40, 41]. The thermal stimulation from hind feet inducing Fos expression in the PVN showed that the PVN can receive the noxious input [42]. The activation of PVN initiates the hypothalamus-pituitary-adrenal hormone cascade by releasing corticotropin-releasing factor (CRF) and arginine vasopressin from its parvocellular cells [43]. Fos expression neurons in those nuclei suggest that injecting bee venom in the groin region activates not only pain transmission pathway but also the nuclei regulating physiological responses and inhibiting pain in the central nervous system. 

### 4.2. Hierarchical Innervation of the Uterus

The PrV transneuronal tracing method is widely used to detect the hierarchically central innervation of urethra, clitoris, penis, urinary bladder, and uterus [23, 24, 44–51]. In our previous study, the Pingtung strain of PrV applied to the auricular kidney point transneuronally and specifically infected sympathetic pre- and postganglionic neurons [22]. In order to investigate the highest central control of uterus, the survival time was proportionally extended to 6–8 days in this study. All PrV-infected nuclei in our study (Table 2) could be found in these nuclei reported by other strains of PrV transneuronal studies [23, 24]. This result confirms that the Pingtung strain of PrV was a sustainable strain as a transneuronal tracer in hierarchical innervation studying.

Lee and Papka discovered PrV-infected sympathetic and parasympathetic preganglionic neurons at T11–13 and L6–S1 spinal segments, after PrV injection in the uterine cervix [23, 24]. The results indicate the visceral efferent of the uterus are mainly from T11–T13 and L6–S1 segments. In our study, PrV-infected preganglionic neurons are mainly in the intermediolateral nucleus (IML) and sacral parasympathetic nucleus of T10–L2 and L6–S1 spinal segments (Figure 2). Our result is in accord with previous studies [23, 24, 50]. 

Supraspinal PrV-infected high hierarchical uterine neurons are located in the NTS, dorsal motor nucleus of vagus (DMX), A5 noradrenaline cell group (A5), raphe pallidus nucleus (RPa), gigantocellular reticular nucleus (Gi), and PVN (Figure 4). The NTS is the major brainstem structure receiving both general and special visceral sensory inputs, including visceral pain [52, 53]. Electrophysiological and HRP studies have shown that the NTS contains neuronal connection with the uterus [54, 55]. The DMX is generally recognized as parasympathetic preganglionic neurons that innervate various visceral organs. Retrograde tracer studies have shown that the DMX innervates the cecum, uterus, and colon directly or indirectly [50, 52, 55–57]. The neurons in the A5 cell group, RPa, and Gi innervating the uterus were also confirmed by previous PrV tracing researches [23, 24, 50]. Therefore, the PVN function is not only a uterus-related hormone regulation center [58], but also a direct neuronal connection to the uterus [23, 24]. After PHA-L injection in the PVN, terminal varicosities appeared in the IML of the thoracic spinal cord [59]. Retrograde tracer studies also showed a neuronal connection between the PVN and the uterus [23, 24, 50]. In summary, all these results suggest that the PVN has either a direct or indirect neuronal connection and it regulates the uterus through both hormonal and neuronal innervations. 

### 4.3. Somatovisceral Neuronal Connection Nuclei between the Groin A-Shi Point and the Uterus Located in the NTS, DMX, and PVN

 Retrograde tracer injection in the NTS suggests that the spinal superficial dorsal horn neurons (lamina I) directly project to the NTS [60]. Our result (Figure 2(a)) and a previous study [35] also showed that the NTS can be activated by somatic noxious stimulation. Several anatomical and electrophysiological studies have shown the neuronal connection between the uterus and NTS and DMX through the vagus nerve [23, 24, 50, 54, 55]. The PrV-infected neurons in the NTS and DMX (Figure 4(a)) also confirmed the efferent innervation of the NTS and DMX to the uterus as in a previous study [55]. Neurons with cell bodies in the DMX send their dendrites into the gelatinosus solitary nucleus, where they receive synaptic inputs from gastric sensory afferents [61]. These researches suggested that the NTS and DMX play important roles in visceral innervation, including the digestive functions of the stomach and baroreceptor reflex [61–66]. The double labeled NTS and DMX neurons in our study suggest that the nuclei may be one of the relay centers of the somatovisceral reflex of the groin A-shi point and uterus. 

Retrograde labeling study shows that the NTS receives spinal projections from the superficial dorsal horn [60] and projects to the PVN [42]. Fos expression studies also suggested that the PVN could be activated by somatic noxious stimulations [42, 67]. Swanson proved that the PVN, a higher hormonal regulation center, projects neuronal fibers to the pituitary gland, medulla, and spinal cord [68]. The neuronal connection between the PVN and the uterus was also proved by previous indicators [23, 24, 50] and our studies (Figure 4(e)). Noxious stimulation and retrograde tracer injection in different visceral organs showed that the PVN was not only receive noxious afferent but also innervate visceral organs as well [24, 69–72]. The reflex inhibition of the heart rate elicited by acupuncture-like stimulation likely occurs through the activation of the hypothalamic nucleus, which inhibits the activity of premotor sympathetic neurons in the rostral ventrolateral medulla (rVLM) [73, 74]. Although bee venom stimulation in groin A-shi point induced less neuronal activity in the PVN, comparing to saline group (Table 1) in our study, there was higher percentage of double labeled neurons in the PVN than saline injection group (Figure 5(d)). Those previous researches and our result suggest that the PVN may be another relay center of the somatovisceral reflex between the groin and uterus.

Although the electrophysiological studies show laminas I and V of the spinal dorsal horn receive afferent information from both somatic and visceral tissues [75–79], no double labeled spinal dorsal neurons can be detected in our study. This solid evidence shows the neuronal connection between the groin region and the uterus is not admitted through the spinal cord.

### 4.4. The Morphologic Evidence of Somatovisceral Reflex: A Possible Neuronal Pathway of Acupuncture

A previous study suggested that acupuncture may influence visceral function via the activation of the somatosensory neurons [9]. However, most researches focused on the physiological responses induced by acupuncture [5, 10, 11]. The purpose of our study is to investigate and provide the morphological evidence of somatovisceral reflex and possible neuronal pathway of acupuncture. Our result suggests that the PVN, NTS, and DMX could be the relay center of the somatovisceral reflex. The visceral organs usually receive sympathetic and parasympathetic dual interacted and the interaction is antagonistic. Therefore, our study proved morphological evidence of both sympathetic and parasympathetic pathways of somatovisceral reflex between the groin A-shi point and the uterus (Figure 6). The somato-parasympathetic pathway starts from the stimulation of the groin A-shi point, which activates neurons in the spinal dorsal horn. The signal in turn elicits noxious input to the NTS [60]. Neurons in the NTS relay the information and project to the DMX [80], which innervates the uterus through the vagus nerve [55] (Figure 6(a)). The somato-sympathetic pathways from the neurons in the spinal dorsal horn project to the NTS [60] then direct connection to the PVN [81]; it innervates the visceral organ through the sympathetic pre- and postganglionic neurons [59] (Figure 6(b)). These complementary somato-sympathetic and somato-parasympathetic systems coincidentally match the concept of Yin-Yang theory in traditional Chinese medicine [2, 82].

In conclusion, the present study provides the morphological evidence of the neuronal connection between somatic groin A-shi point and its corresponding visceral organ uterus. Therefore, we come up to the conclusion that the somato-sympathetic/somatoparasympathetic pathways are the morphological basis of somatovisceral reflex and also the neuronal substrate of acupuncture pathways. 

## Figures and Tables

**Table 1:** Fos expression neurons in supraspinal area between saline and bee venom injection groups.

c-fos expression neurons in supraspinal area	Saline	Bee venom
Diencephalon		
Thalamus		
Paratenial thalamic nucleus (PT)	96.3 ± 49.5	105.8 ± 27.2
Precommissural nucleus (PrC)	69.6 ± 18.6	53.2 ± 17.4
Rhomboid thalamic nucleus (Rh)	44.9 ± 21.1	62.8 ± 15.2
Reuniens thalamic nucleus (Re)	112.6 ± 36.0	88.9 ± 24.3
Pretectal nucleus	89.9 ± 18.6	61.3 ± 16.0
Lateral dorsal thalamic nucleus (LD)	83.4 ± 25.9	72.6 ± 21.9
Mediodorsal thalamic nucleus (MD)	134.7 ± 56.9	163.5 ± 47.6
Paraventricular thalamic nucleus (PV)	98.9 ± 28.9	75.9 ± 17.8
Lateral habenular nucleus (LHb)	37.8 ± 5.0	44.4 ± 13.0
Lateral posterior thalamic nucleus (LP)	82.9 ± 30.6	89.5 ± 29.9
Lateral geniculate nucleus (LG)	82.7 ± 23.5	62.9 ± 13.3
Suprageniculate thalamic nucleus (SG)	97.0 ± 40.4	72.1 ± 18.2
Hypothalamus		
Medial preoptic area (MPA)	82.3 ± 17.2	94.9 ± 24.9
Suprachiasmatic nucleus (SCh)	67.0 ± 15.8	72.3 ± 13.7
Arcuate nucleus (Arc)	50.4 ± 15.3	50.8 ± 11.6
Supramammillary nucleus (SuM)	172.6 ± 17.3	189.2 ± 33.3
Lateral mammillary nucleus (LM)	94.3 ± 13.2	92.6 ± 29.0
Supraoptic nucleus (SO)	41.0 ± 19.5	86.7 ± 20.6
Lateral hypothalamic area (LH)	108.6 ± 20.3	121.0 ± 26.2
Premammillary nucleus (PM)	136.4 ± 13.0	96.1 ± 40.0
Posterior hypothalamic area (PH)	169.4 ± 33.1	149.4 ± 28.6
Ventromedial hypothalamic nucleus (VMH)	100.1 ± 44.8	86.9 ± 36.4
Dorsomedial hypothalamic nucleus (DM)	142.2 ± 30.2	150.4 ± 25.0
Anterior hypothalamic area (AHC)	100.1 ± 30.6	73.9 ± 15.6
Lateroanterior hypothalamic nucleus (LA)	118.4 ± 19.4	133.2 ± 55.5
Paraventricular hypothalamic nucleus (PVN)	82.3 ± 14.3	62.1 ± 9.7
Anterodorsal preoptic nucleus (ADP)	60.3 ± 20.3	60.9 ± 8.8
Mesencephalon		
Edinger-Westphal nucleus (EW)	30.7 ± 4.2	29.9 ± 3.1
Paranigral nucleus (PN)	51.6 ± 14.9	61.0 ± 13.2
Interfascicular nucleus (IF)	26.1 ± 6.6	30.4 ± 4.6
Pons		
Parabrachial nucleus (PB)	70.1 ± 15.9	52.6 ± 13.1
Dorsal raphe nucleus ventrolateral part (DRVL)	52.6 ± 35.8	90.6 ± 17.2
Dorsal raphe nucleus (DR)	37.3 ± 16.1	16.1 ± 4.3
Pontine nuclei (Pn)	277.1 ± 107.9	298.7 ± 68.2
Nucleus of lateral lemniscus (LL)	58.0 ± 22.7	64.8 ± 12.0
Locus coeruleus (LC)	73.4 ± 14.8	31.4 ± 5.7
Prepositus nucleus (Pr)	13.7 ± 8.0	22.3 ± 6.4
Area postrema (AP)	21.8 ± 8.3	31.4 ± 11.9
Medulla		
Gigantocellular reticular nucleus (Gi)	16.4 ± 2.2	14.7 ± 4.9
Raphe magnus nucleus (RMg)	20.6 ± 1.7	11.9 ± 3.3
Raphe pallidus nucleus (RPa)	12.6 ± 2.3	19.1 ± 4.8
Medial vestibular nucleus (MVe)	33.5 ± 3.9	45.7 ± 7.1
Inferior olivary nucleus (IO)	40.7 ± 10.7	39.5 ± 8.2
Caudoventrolateral reticular nucleus (CVL)	18.8 ± 4.9	18.9 ± 3.9
Nucleus of solitary tract (NTS)	17.1 ± 4.0	33.8 ± 5.0*

*Significant difference between saline and bee venom injection groups, *P* < 0.1.

**Table 2:** PrV-infected neurons in supraspinal area.

PrV-infected neurons in supraspinal area	
Hypothalamus	
Medial preoptic area (MPA)	+
Arcuate nucleus (Arc)	+
Ventromedial hypothalamic nucleus (VMH)	+
Dorsomedial hypothalamic nucleus (DM)	+
Paraventricular hypothalamic nucleus (PVN)	++
Pons	
Locus coeruleus (LC)	+
Medulla	
Gigantocellular reticular nucleus (Gi)	+++
Raphe magnus nucleus (RMg)	++
Raphe pallidus nucleus (RPa)	+
Caudoventrolateral reticular nucleus (CVL)	++
Nucleus of solitary tract (NTS)	+++
Dorsal motor nucleus of vagus (DMV)	+++
A5 noradrenaline cells (A5)	++
Lateral reticular nucleus (LRt)	++

+: 1–3 infected neurons, ++: 4–8 infected neurons, and +++: >9 infected neurons.

## Abbreviations

PrV: Pseudorabies virus
NTS: Nucleus of solitary tract
PVN: Paraventricular hypothalamic nucleus
DMX: Dorsal motor nucleus of vagus
PB: Parabrachial nucleus
LC: Locus coeruleus
RPa: Raphe pallidus nucleus
PVT: Paraventricular thalamic nucleus
LH: Lateral hypothalamic area
IRt: Intermediate reticular nucleus
Amb: Ambiguus nucleus
LRt: Lateral reticular nucleus
A5: A5 noradrenaline cells group
Gi: Gigantocellular reticular nucleus
MPA: Medial preoptic area
CRF: Corticotropin-releasing factor
IML: Intermediolateral nucleus
SPN: Sympathetic preganglionic neurons.
